# Molecular mechanisms of bacteria-mediated cancer immunotherapy: from intratumoral microbiota to engineered therapeutics

**DOI:** 10.3389/fimmu.2026.1878878

**Published:** 2026-07-28

**Authors:** Jinhu Guo, Yimeng Li, Yue Yang, Bobo Wang

**Affiliations:** Yan’an Medical College, Yan’an University, Yan’an, Shaanxi, China

**Keywords:** bacteria-mediated cancer immunotherapy, cancer, immunotherapy, intratumoral microbiota, tumor-immune interface

## Abstract

Immunotherapy has redefined oncology, yet its efficacy remains constrained by low response rates, primary or acquired resistance, immune-related toxicities, and escalating costs. Bacteria-mediated cancer immunotherapy (BCIT), which exploits the intratumoral microbiota as a programmable immunotherapeutic platform, has therefore emerged as a promising strategy. Although anecdotal links between infection and tumor regression were documented over four millennia ago, the molecular underpinnings of BCIT have only recently become accessible through synthetic biology, single-cell sequencing, and gnotobiotic modeling. Here we synthesize current knowledge on how intratumoral bacteria either enhance or suppress malignancy via genotoxicity, epigenetic reprogramming, metabolic competition, and modulation of the tumor-immune interface. We dissect cutting-edge engineering approaches—quorum-sensing circuits, thermo-inducible switches, molecular mimicry, and biohybrid microrobots, that convert commensal or attenuated pathogenic strains into precision delivery vehicles for cytokines, checkpoint inhibitors, and neoantigens. Finally, we critically evaluate translational bottlenecks (safety, pharmacokinetics, regulatory science, inter-patient heterogeneity) and propose an AI-guided, microbiome-integrated framework to accelerate clinical translation. This Review provides a conceptual framework for harnessing living therapeutics to convert immunologically “cold” tumors into “hot”, therapy-sensitive lesions, and discusses key directions for future microbiome-driven oncology trials.

## 1 Introduction

Progress in the field of cancer immunotherapy is enhancing the potential for curative outcomes and broadening the spectrum of cancers that respond to these innovative therapeutic approaches, thereby instilling renewed optimism among patients (1). However, many cancers still do not respond to immunotherapies. For example, 40-65% of melanoma patients and 45-85% of non-small-cell lung cancer (NSCLC) patients have innate resistance to immune-checkpoint inhibitors (ICIs), despite these tumors being among the most responsive (2–5). This underscores the need for more effective treatment strategies to tackle resistance.

The human microbiota significantly impacts tumorigenesis and cancer therapy (6–8). While gut microbiota research dominates these fields, emerging insights highlight the existence and pathological importance of intratumoral microbiota (9–12). Recent research has shown that bacteria living in tumors are not just passive observers but actively contribute to cancer development and change the immune environment within tumors. However, the impact of these bacteria varies contextually: while certain bacteria facilitate tumor progression, others possess the capacity to suppress it. This intricate interplay between the microbiota and the immune system in cancer patients presents novel therapeutic avenues.

The study of intratumoral microbiota has gained momentum thanks to advancements in sequencing, imaging, machine learning, *in vitro* culture, and the application of genetically engineered and germ-free mouse models (11, 13–18). In particular, next-generation sequencing has significantly enhanced understanding of the microbiome’s diversity and its functional importance in solid tumors (13, 14, 16, 19–21). 16S rRNA sequencing serves as the conventional approach for classifying bacteria into distinct taxonomic groups. In contrast, shotgun metagenomic DNA sequencing provides an in-depth analysis of entire microbial genomes, shedding light on both the taxonomic classification and functional dimensions of the intratumoral microbiome (16). However, the underlying reasons for some microorganisms facilitating carcinogenesis while others boost anticancer immune reactions remain to be fully elucidated.

**Figure 1:**
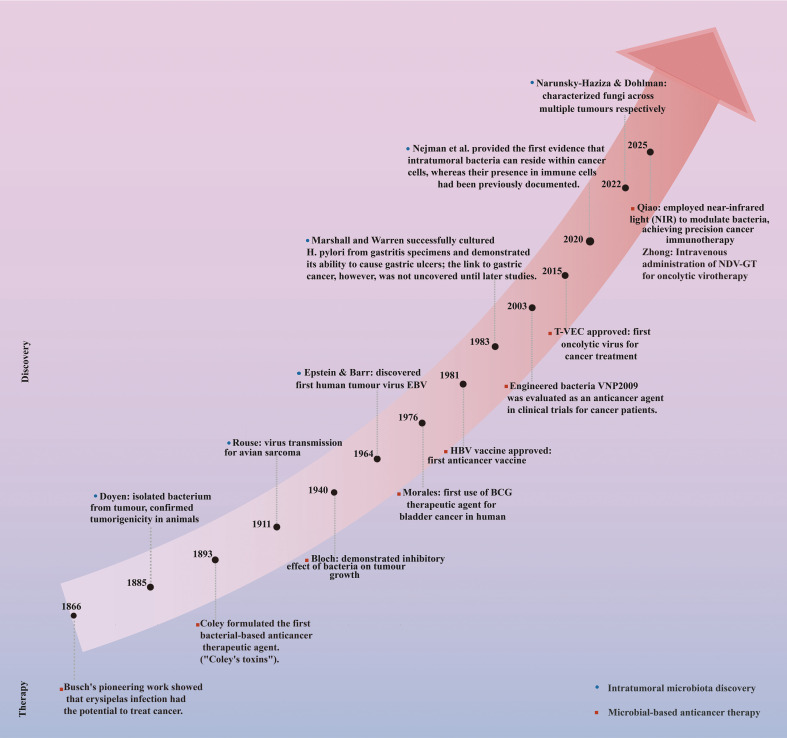
Historical milestones in intratumoral microbiota research and bacteria-based cancer therapy. This timeline summarizes key discoveries and therapeutic applications spanning from the 19th century to the present. Events are organized into two parallel streams: Basic Research & Mechanistic Discoveries (left) and Therapeutic Applications & Clinical Milestones (right). Major breakthroughs are highlighted with larger icons, whereas supporting findings are shown with smaller markers. Key milestones include the first observation of bacteria-associated tumor regression (Busch, 1866), the development of Coley's toxin (1893), the first clinical use of BCG for bladder cancer (Morales, 1976), the discovery of H. pylori (Marshall & Warren, 1983), the landmark characterization of tumor-type-specific intratumoral microbiomes (Nejman et al., 2020), and the approval of the first oncolytic virus T-VEC (2015). This figure provides a historical framework for the subsequent mechanistic and engineering discussions in this Review. Created with Adobe Illustrator 2023.

Numerous significant findings regarding intratumoral microbiota have been documented to date (**Figure 1**). Over the past two decades, there has been increasing interest in utilizing live bacteria for cancer immunotherapy, a concept that originated 4,000 years ago (22). These live bacteria possess unique attributes that distinguish them from traditional anticancer agents (23–25). Some bacteria can home in on cancer tissues with specificity, infiltrate tumors deeply, proliferate rapidly, and reprogram the immune microenvironment of “cold” tumors to “hot” tumors (26). Additionally, bacteria can be tailored to serve as adaptable drug delivery systems (23).

In this Review, we detail the diverse characteristics, functions, and interactions of bacteria within the TME. We analyze the role of intratumoral bacteria in both promoting and inhibiting tumor development, and we explore the implications for patient management. Particular attention is given to the application of specific bacterial strains and engineering methods in bacteria-mediated cancer immunotherapy (BCIT), including bacteria-based *in situ* vaccines and biorobots. Finally, we delve into the molecular mechanisms of bacteria in cancer immunotherapy and discuss potential future directions and challenges in this emerging field of research.

## 2 Neoplastic transformation and tumor evolution

**Figure 2:**
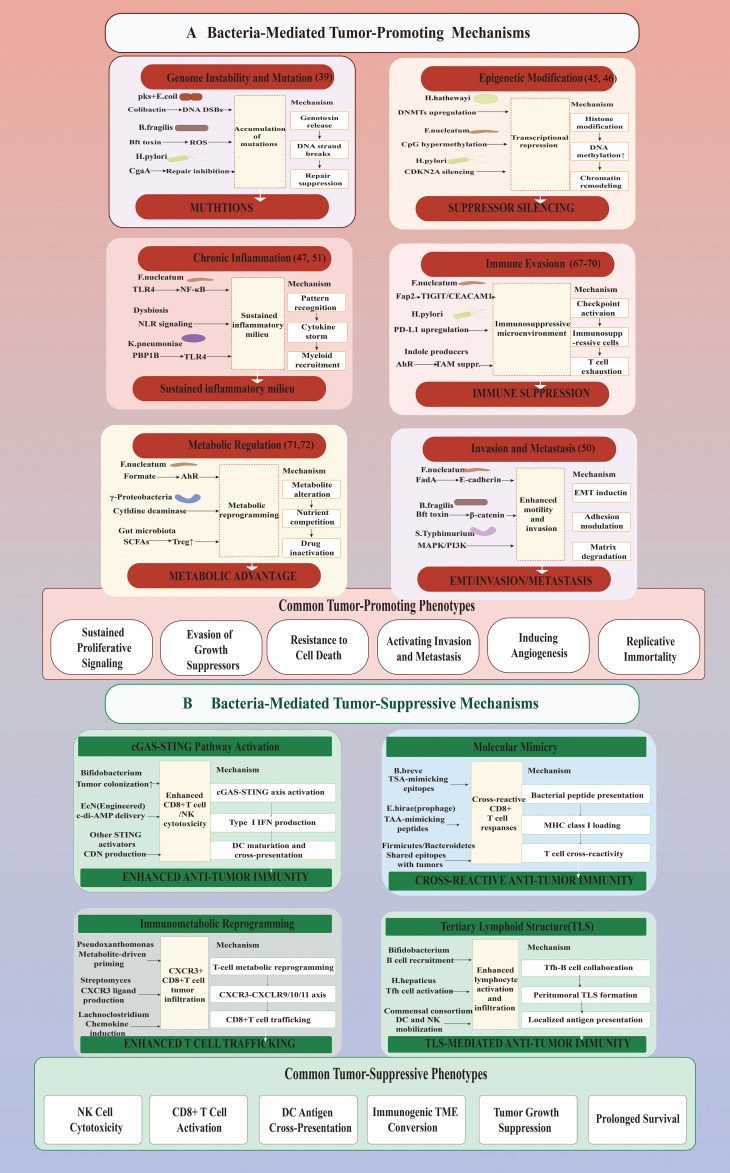
Proposed mechanisms by which intratumoral microbiota contribute to cancer development. This schematic illustrates six interconnected pathways through which tumor-resident bacteria may drive oncogenesis and tumor progression: (1) genome instability and mutation (via genotoxins such as colibactin); (2) epigenetic modification (e.g., promoter hypermethylation of tumor-suppressor genes); (3) chronic inflammation (via NF-κB activation and IL-23/IL-17 signaling); (4) immune evasion (via PD-L1 upregulation and recruitment of immunosuppressive cells such as MDSCs and Tregs); (5) metabolic reprogramming (e.g., HK2-mediated glycolysis and TCA cycle alterations); and (6) activation of invasion and metastasis (via epithelial–mesenchymal transition and matrix remodeling). The flow depicts a causal chain from bacterial factors (left) to host signaling pathways (center) to tumor phenotypic outcomes (right). Abbreviations: TAM, tumor-associated macrophage; HK2, hexokinase 2; TCA, tricarboxylic acid. Created with Adobe Illustrator 2023.

For more than a century, research has indicated that bacteria can exist within tumor tissues (13, 27). However, in 2020, a groundbreaking study examining the bacterial composition across seven types of cancer revealed that these microorganisms can have opposing functions within tumors (13). It is now well-established that bacteria residing within tumors can either promote or inhibit cancer development. However, the specific molecular mechanisms underlying bacterial interactions with cancer cells or TME components remain poorly understood. Cancer cells drive disease progression through mechanisms such as sustained proliferation, evasion of growth inhibition, resistance to cell death, replicative immortality, induction of angiogenesis, and activation of invasion and metastasis. Although the role of the microbiome in cancer initiation and progression is not yet fully understood, it may influence tumor-promoting interactions between malignant and non-malignant cells. Clarifying these mechanisms is crucial for cancer prediction and treatment (**Figure 2**).

Origins of intratumoral bacteria and their potential functional implications. Three potential sources have been proposed: (i) breaching of mucosal barriers during oncogenesis; (ii) pre-existing bacteria in adjacent non-tumor tissues; and (iii) hematogenous spread from distant mucosal sites such as the oral cavity or gut (28). While these origins are plausible, the field lacks direct evidence linking a specific source to distinct immunological functions, tumor tropism, or therapeutic implications. This represents a critical knowledge gap.

One might hypothesize that bacteria from breached mucosal barriers (e.g., Fusobacterium nucleatum in colorectal cancer) preferentially engage with epithelial Toll-like receptors and drive chronic inflammation, whereas hematogenously delivered bacteria (e.g., Salmonella in pancreatic cancer) may reside intracellularly within immune cells and modulate antigen presentation. Bacteria from adjacent non-tumor tissues could represent a “field effect” where pre-existing commensals are gradually co-opted into the tumor niche. However, these hypotheses remain speculative.

Distinguishing between these origins is methodologically challenging because bacteria from all three sources ultimately converge within the tumor. Future studies using gnotobiotic mouse models with barcoded bacterial libraries are needed to trace the fate and function of bacteria from each compartment. Until such data are available, the field should avoid assuming functional equivalence among bacteria from different origins. Studies employing preclinical models and clinical samples have shown that intratumoral bacteria mainly inhabit the cytoplasm of immune or cancer cells and remain viable (13, 29). These findings indicate that the intratumoral microbiota is probably an integral and inherent component of tumors, rather than just a secondary result of pathogenic infections (29). Comprehensive reviews detailing the intratumoral microbiome across various cancer types are available in other publications (10, 16, 18, 30). Herein, we primarily focus on the molecular mechanisms underlying the exploitation of bacteria for cancer immunotherapy.

### 2.1 Oncogenesis and cancer progression

**Figure 3:**
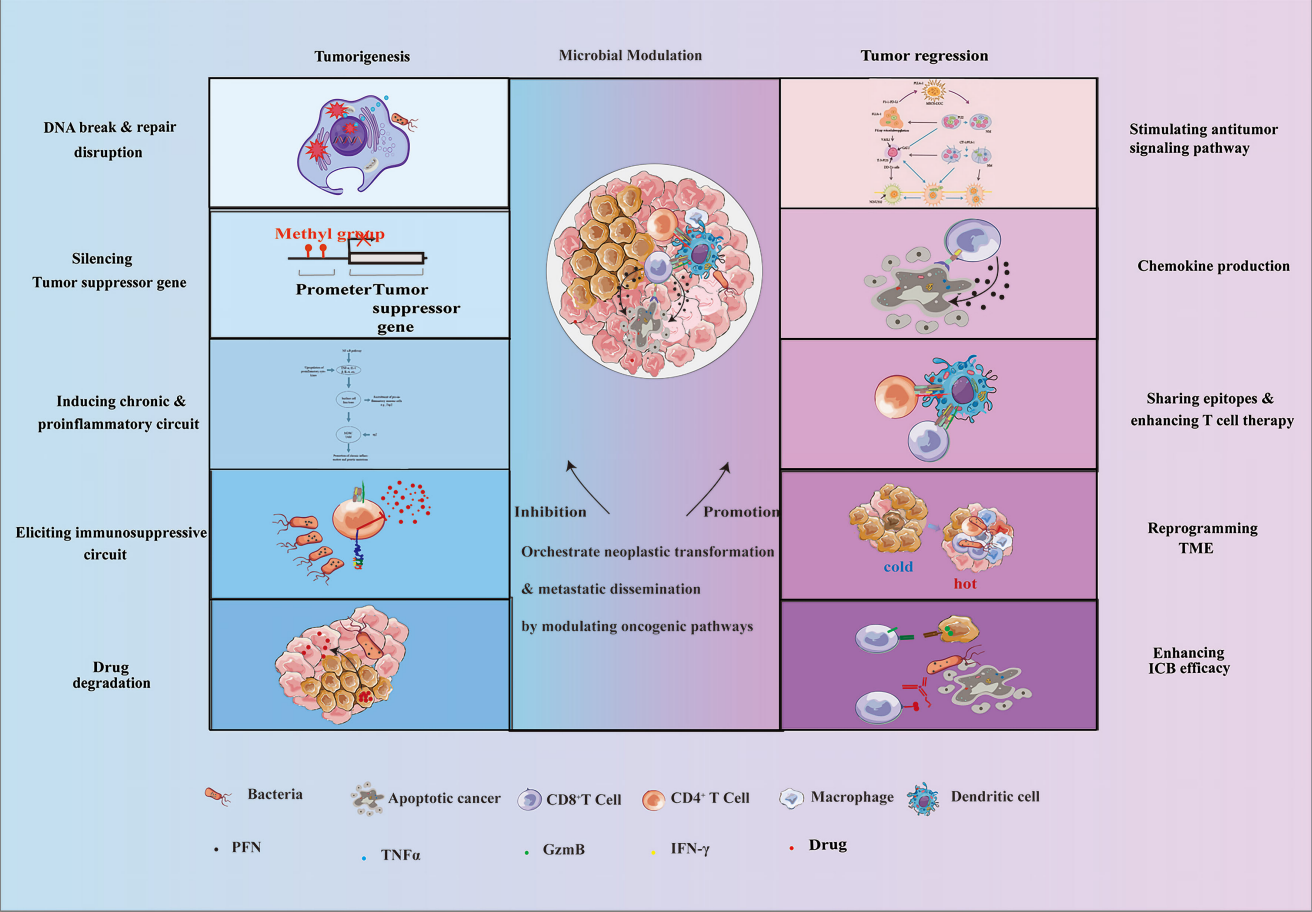
Dual mechanisms of intratumoral bacteria in tumor promotion (left) and suppression (right). This figure contrasts the pro-tumorigenic and anti-tumorigenic functions of tumor-resident bacteria. The left panel depicts tumor-promoting mechanisms: (1) genotoxicity (DNA double-strand breaks and repair disruption); (2) epigenetic silencing (promoter hypermethylation of tumor-suppressor genes); (3) chronic proinflammatory signaling (NF-κB, IL-23/IL-17); (4) immunosuppression (MDSC/Treg recruitment and PD-L1 upregulation); and (5) chemoresistance via drug degradation or autophagy induction.The right panel illustrates tumor-suppressive mechanisms: (1) activation of anti-tumor signaling (STING/type I IFN pathway); (2) chemokine production and CD8^+^ T cell infiltration; (3) molecular mimicry enabling T cell cross-reactivity; (4) remodeling of the immunosuppressive TME ("cold" to "hot"); and (5) synergistic enhancement of ICB efficacy.The central bridging column highlights bacterial effectors and metabolites that mediate these opposing outcomes, including genotoxins, adhesins, LPS, and flagellin. Abbreviations: GzmB, granzyme B; TNFα, tumor necrosis factor α; IFNγ, interferon γ; PFN, perforin; MDSC, myeloid-derived suppressor cell; Treg, regulatory T cell; ICB, immune checkpoint blockade. Created with Adobe Illustrator 2023.

Recent studies have further elucidated the functional diversity of intratumoral microbiota across various cancer types, highlighting their diagnostic and therapeutic potential, and have explored advanced strategies to modulate these microbial communities for improved therapeutic outcomes (31–34). The carcinogenic potential of specific microbial consortia has been mechanistically linked to neoplastic development across diverse anatomical compartments including mammary glands, osseous structures, pulmonary parenchyma, hepatic tissues, pancreatic ducts, ovarian stroma, renal epithelia, melanocytic lesions, cervical mucosa, cerebral microenvironments, and gastrointestinal ecosystems, thereby functioning as pathobiont-mediated oncogenic drivers (9, 35, 36). These microbial entities mediate neoplastic transformation and metastatic dissemination by modulating interconnected oncogenic pathways within tumor ecosystems (9, 10, 30, 35) (**Figure 3**).

Bacteria demonstrate carcinogenic potential through modulation of host genome fidelity. Particular bacterial species secrete low-molecular-weight genotoxic metabolites capable of inducing double-strand breaks and heritable genetic alterations in pre-malignant epithelial populations (37–40). For instance, certain enteric bacterial strains, like *pks^+^ Escherichia coli*, generate the complex secondary metabolite colibactin. This compound alkylates and crosslinks DNA bases, resulting in DNA double-strand breaks (DSBs) (39). Data from mouse models suggest these bacteria are involved in inflammation-associated colorectal cancer (CRC) tumorigenesis (39). Bacterial toxins including *Bacteroides fragilis* enterotoxin (Bft) enhance the generation of reactive oxygen species. As a result, DNA undergoes base oxidation and strand breakage, processes that are pivotal in facilitating CRC development in mouse models (9, 41, 42). Additionally, certain bacteria, such as *Chlamydia trachomatis* linked to gynecological cancers and *Helicobacter pylori* associated with gastric cancers, can influence the host’s DNA repair mechanisms. These bacteria achieve this by suppressing homologous recombination, thereby enabling the error-prone non-homologous end-joining process and consequently leading to an elevated number of errors in DSB repair (43, 44). *C. trachomatis* inhibits the activation of ATM, a crucial DNA damage response (DDR) mediator, by blocking its release from PP2A (44). Consequently, this action disrupts the homologous recombination repair process (44). *H. pylori* increases the expression of small nuclear RNA host gene 17 (SNHG17), leading to a shift in DSB repair from homologous recombination towards non-homologous end-joining (43). Bacteria such as *Hungatella hathewayi* and *Fusobacterium nucleatum*, associated with CRC development, induce epigenetic changes in the host genome by upregulating DNA methyltransferases through unknown mechanisms (45, 46). This results in hypermethylation and silencing of tumor-suppressor genes including *CDX2* and *MLH1*, driving tumorigenesis (45, 46). Multiple bacterial effectors and adhesins activate cancer-promoting signaling pathways. For example, the *H. pylori* cytotoxin-associated gene A (CagA) protein not only promotes the hypermethylation of tumor-suppressor genes such as *CDKN2A* but also contributes to gastric cancer tumorigenesis by interacting with E-cadherin and dysregulating β-catenin signaling in host epithelial cells (47). Similarly, the binding of *F. nucleatum’*s adhesin FadA to E-cadherin triggers β-catenin’s nuclear translocation, fostering CRC development (48). Bft drives E-cadherin ectodomain cleavage, β-catenin’s nuclear relocation, and IL-8 secretion, stimulating cancer cell proliferation (49). Various effector proteins from *Salmonella enterica* serovar Typhimurium (*S.* Typhimurium) and *S. enterica* serovar Typhi (*S.* Typhi) can also activate the MAPK-ERK and PI3K-AKT-mTOR pathways, driving gallbladder cancer cellular transformation (50).

The longitudinal maintenance of microbiota-triggered immunopathological states establishes permissive niches for oncogenic transformation via spatiotemporal crosstalk between myeloid cells and dysbiotic microbiota (9, 30). Various cancer-associated bacteria, such as *F. nucleatum*, can bind to pattern recognition receptors like Toll-like receptors (TLRs), leading to the activation of NF-κB, a key transcription factor that drives inflammatory responses (51). Yet, other pattern recognition receptors, such as *NOD2*, NLRP3, *NLRP6*, and *NLRP12* within the nucleotide-binding oligomerization domain-like receptor (NLR) family, serve as negative regulators of inflammatory responses and can block the progression of colitis-associated CRC (52–57). Longitudinal investigations in inflammation-associated colorectal carcinogenesis models demonstrate that combinatorial perturbations of host defense systems—including NLR-mediated pattern recognition deficits and genotoxic stress under inflammatory milieus—selectively amplify bacterial lineages with heightened capacities for mucosal colonization, parenchymal invasion, and inflammasome activation (52–57). For instance, *Nod2* or *Nlrp6*-deficient mice develop transmissible gut microbial communities that increase susceptibility to colitis-associated CRC in both themselves and their wild-type littermates (55, 58). Spatiotemporal profiling identifies that microbiota-encoded CCL5/IL-6 crosstalk generates field cancerization effects through JAK-STAT-mediated epithelial reprogramming, establishing molecular trajectories toward adenocarcinoma progression in predisposed mucosae (59).

In the TME, bacterial activation of innate immune cells drives adaptive immune responses, such as via IL-23-IL-17 axis activation (60), TNF receptor signaling (61, 62), IL-6 signaling (63), and/or STAT3 signaling (64). Genetic dissection of *Apc*-driven intestinal tumorigenesis reveals that hematopoietic MyD88 mediates tumor niche-specific IL-6/IL-17A co-amplification, whereas gut microbial depletion through combinatorial antibiotic intervention disrupts the IL-23-driven inflammatory-immune axis, leading to dual suppression of IL-17A/STAT3 circuit activation within neoplastic clones (60). In conclusion, integrative analyses position microbiota-directed immune surveillance as a dual-edged modulator of neoplastic advancement, while mechanistic studies identify resident pulmonary microbial communities as instigators of protumorigenic cytokine storms through pattern recognition receptor crosstalk in preclinical bronchogenic carcinoma systems. This occurs as these bacteria stimulate myeloid cells to secrete IL-1β and IL-23, which in turn activate IL-17-producing γδ T cells that are tissue-resident (65). Depletion of commensal microbiota via sterile husbandry or β-lactam-based eradication protocols attenuates KrasG12D-mediated pulmonary adenocarcinoma progression, demonstrating that microbial metabolite-driven STAT3/IL-17 signaling circuits constitute a fundamental axis linking symbiotic homeostasis to oncogenic niche formation.

Intratumoral bacteria activate pro-inflammatory immune responses and induce immunosuppressive reactions. *F. nucleatum* adhesins, including Fap2 and CbpF, interact with inhibitory receptors such as TIGIT on natural killer (NK) cells and CEACAM1 on T cells. This interaction may inhibit the infiltration and activation of these immune cells within the TME of CRC and pancreatic ductal adenocarcinoma (PDAC) (66). In addition, this bacterium selectively attracts myeloid-derived suppressor cells (MDSCs) to tumors in CRC mouse models, inducing immunosuppression within the TME and facilitating metastasis (67). *H. pylori* and *S. Typhimurium* upregulate the expression of the immune checkpoint protein PD-L1 in various host cells to avoid immune detection (68, 69), a mechanism that may enhance immune evasion in tumors associated with these bacteria. Bacterial metabolites, such as indole compounds derived from tryptophan by *Lactobacillus*, can suppress anticancer immunity. They achieve this by activating immunosuppressive TAMs through aryl hydrocarbon receptor (AhR) signaling, which impairs anti-tumor responses of CD8^+^ T cells in PDAC mouse models (70). Formate, a metabolite produced by *F. nucleatum*, activates AhR signaling and promotes CRC development in mouse models (71). This is partly due to enhanced inflammatory responses from Th17 cell expansion. In patients with cancer, elevated serum levels of short-chain fatty acids like butyrate and propionate, derived from gut bacteria, are linked to higher circulating regulatory T (T_reg_) cell proportions among CD4^+^ T cells and resistance to anti-CTLA-4 antibodies (72).

### 2.2 Tumor regression

#### 2.2.1 Enhanced anticancer immunity

Tumor-resident microbes can shape the immune microenvironment by modulating local immune cell cytokines and activities, yet their impact on anti-tumor immunity is context-dependent, as they may either drive or suppress Tumor growth. Spatiotemporal analyses reveal that microbiota-immune crosstalk mediates a feedforward loop of oncogenic niche formation through epigenetic reprogramming of tumor-infiltrating lymphocytes.

The dominance of *Pseudoxanthomonas*, *Streptomyces*, *Saccharopolyspora*, and *Bacillus clausii* in PDAC-associated microbial networks correlates with prolonged patient survival, suggesting synergistic bacterial activities may enhance anti-tumor immunity through metabolite-driven T-cell priming (21). Notably, fecal microbiota transplantation (FMT) from these long-term survivors altered the gut and tumor microbiome in mice, stimulating CD8^+^ T cell-driven anticancer responses and curbing tumor growth (21). These findings establish a dynamic crosstalk between tumor-resident microbiota and the enteric microbiome, suggesting the presence of oncosuppressive microbial niches within tumors. Although direct bacteriolytic effects remain rarely documented, preclinical evidence indicates that specific taxa (e.g., *Proteus mirabilis* and *Cutibacterium acnes*) and their polymicrobial communities drive syngeneic tumor regression through immune-contexture remodeling, notably by reprogramming immunosuppressive microenvironments (73, 74).

Tumor-resident microbiota enhances anticancer immune responses by orchestrating cytosolic DNA sensing via the cGAS-interferon genes (STING) axis, augmenting cytolytic activity of lymphoid lineages (CD8^+^/NK cells), and facilitating ectopic germinal center formation with enhanced cross-presentation capacity (30). *Bifidobacterium* colonizing neoplastic niches mediate cGAS-STING axis engagement, amplify dendritic cells (DCs)-mediated epitope cross-presentation, and enhance anti-CD47-driven tumoricidal efficacy through immunometabolic reprogramming in murine systems (12). In addition, *Saccharopolyspora-Pseudoxanthomonas-Streptomyces* consortia colonizing PDAC niches drive immunometabolic reprogramming that amplifies CXCR3-dependent CD8^+^ T cell trafficking and effector differentiation, leading to prolonged host survival in murine systems (21). Correspondingly, enrichment of *Lachnoclostridium* within tumors correlates with elevated chemokine levels, increased infiltration of CD8^+^ T lymphocytes in cutaneous melanoma lesions, and improved survival outcomes (75). In murine models of colon cancer, *Helicobacter hepaticus* elicits anti-tumor immunity through the activation of T follicular helper cells, B lymphocytes, and NK cells; this protective effect is mechanistically linked to the formation of peritumoral tertiary lymphoid structures mediated by *H. hepaticus*-reactive T follicular helper cells (76). Furthermore, tumor cells presenting peptides originating from intratumoral bacteria can stimulate tumor-targeting T cell activity (77). This large-scale profiling study encompassing 1,526 tumors from seven distinct malignancies revealed multiple statistically significant and mechanistically interpretable links between bacterial taxa, specific cancer types, and immune cell populations (13).

#### 2.2.2 Molecular mimicry between cancer and bacterial antigens

Malignant cells frequently express diverse aberrant neoantigens, rendering them immunologically foreign. These epitopes undergo presentation via MHC complexes for cognate T cell recognition. Tumor antigens stratify into two principal classes: tumor-associated antigens (TAAs), exhibiting elevated expression in malignancies yet constitutively present in select healthy tissues; and tumor-specific antigens (TSAs), exclusively generated by somatic mutations in transformed cells. Crucially, TSA-mimicking peptide epitopes have been identified within *Bifidobacterium breve* (78) and prophage-containing *Enterococcus hirae* (79). Epitope similarity facilitates T cell cross-reactivity, amplifying anti-tumor immunity and therapeutic efficacy in preclinical models. Furthermore, substantial structural and linear homology exists between TAAs and peptides derived from dominant gut microbiota phyla (Firmicutes, Bacteroidetes) (80). Such molecular mimicry indicates that antimicrobial CD8^+^ T cells may engage TAAs on malignant cells, implying that cross-reactive responses exert fundamental roles in tumor immunosurveillance (80, 81).

### 2.3 Effects of intratumoral bacteria on cancer therapy

Certain tumor-resident microbiota correlate with chemoresistance (22) (**Figure 3**). In PDAC, intratumoral Gammaproteobacteria expressing cytidine deaminase confer gemcitabine resistance through enzymatic drug degradation (82). Similarly, oral pathogens such as *Aggregatibacter actinomycetemcomitans*—implicated in PDAC risk—exhibit analogous resistance mechanisms via identical enzyme expression (83). Clinical evidence further associates biliary stent implantation with Enterobacterales colonization in PDAC tumors (84), potentially elevating resistance to gemcitabine and paclitaxel. For CRC patients, *Fusobacterium nucleatum* enhances 5-fluorouracil and oxaliplatin resistance by TLR4-MYD88 pathway-mediated autophagy induction, achieved through selective downregulation of miR-18a* and miR-4802 (85).

While intratumoral microbiota’s role in recurrence remains incompletely characterized, colon cancer patients exhibiting Clostridiales-enriched tumors demonstrate increased recurrence risk (86).

Paradoxically, *F. nucleatum* presence in oral squamous cell carcinomas predicts superior recurrence-free survival, metastasis-free survival, and overall survival compared to *F. nucleatum*-negative cases (87).

Some intratumoral bacteria can boost immunotherapy efficacy (12). For instance, in mouse models of colon cancer or lymphoma, *Bifidobacterium* species accumulating in the tumor enhance anti-CD47 antibody activity by modulating STING and interferon signaling (12).

Similarly, giving mice with 4T1 breast tumors *Megasphaera* species, linked to longer survival in Chinese PDAC patients, boosted anti-PD-1 antibody anti-tumor efficacy (88). Conversely, antibiotics eliminating PDAC microbiota in mouse models caused immunogenic TME reprogramming, reducing MDSCs and upregulating PD-1 on CD4^+^ and CD8^+^ T cells, thus enhancing anti-PD-1 antibody sensitivity (89). Consequently, co-administration of oral antibiotics with checkICIs represents a viable treatment approach. Analysis of metastatic melanoma patients revealed baseline tumor enrichment of *Clostridium* in ICI responders, whereas non-responders exhibited *Gardnerella vaginalis* enrichment (13). This indicates that microbiota manipulation might enhance immunotherapeutic efficacy.

Collectively, these results indicate the potential advantages of assessing the intratumoral microbiota before treatment. Strategic suppression of chemotherapy-compromising microbiota via antibiotics or introduction of engineered microbial consortia into the TME may enhance therapeutic efficacy. Opportunities also exist for precision microbiome editing through advanced biotechnological approaches, including the deployment of bacteriophages and antagonistic microbial interference strategies (90–92). Nevertheless, developing tumor-type-specific microbiota signatures demands more rigorous investigation. To preserve indigenous microbiota equilibrium, clinical antibiotic administration warrants judicious minimization—restricted exclusively to minimal efficacious regimens essential for therapeutic benefit. Key examples of naturally occurring intratumoral bacteria and their dual roles are summarized in **Table 1**.

**Table 1:** Naturally occurring intratumoral bacteria and their dual roles in tumor promotion and suppression.

Bacterial taxa	Cancer type	Tumor-promoting / suppressive	Main mechanism	Immune cell involvement	Representative references	Primary evidence
*pks^+^ Escherichia coli*	Colorectal cancer (CRC)	Promoting	Colibactin → DNA double-strand breaks	–	(39)	Murine models + human CRC cohorts
*Bacteroides fragilis* (ETBF)	CRC	Promoting	Bft toxin → ROS; E-cadherin cleavage → β-catenin	IL-8 secretion	(9, 41, 42, 49)	Murine models + human CRC cohorts
*Fusobacterium nucleatum*	CRC, PDAC, oral SCC	Dual	FadA → β-catenin; Fap2/CbpF → TIGIT/CEACAM1; MDSC recruitment	T cells, NK cells, MDSCs	(48, 66, 67, 85, 87)	Murine models + patient cohorts (CRC, PDAC, OSCC)
*Helicobacter pylori*	Gastric cancer	Promoting	CagA → β-catenin, DNA repair inhibition	PD-L1 upregulation	(43, 47, 69)	Human epidemiological & mechanistic studies
*Chlamydia trachomatis*	Gynecological cancers	Promoting	Inhibits ATM → impairs homologous recombination	–	(44)	*In vitro* cell models
*Salmonella enterica (Typhimurium/Typhi)*	Gallbladder cancer	Promoting	Effector proteins → MAPK-ERK, PI3K-AKT-mTOR	PD-L1 upregulation	(50, 68)	*In vitro* + murine models
*Hungatella hathewayi*	CRC	Promoting	Upregulates DNA methyltransferases → hypermethylation	–	(45)	Human CRC tissue analysis
*Pseudoxanthomonas + Streptomyces + Saccharopolyspora + Bacillus clausii consortium*	PDAC	Suppressive	Metabolite-driven T-cell priming; CXCR3-dependent CD8^+^ T cell trafficking	CD8^+^ T cells	(21)	Patient cohorts + FMT murine models
*Bifidobacterium* spp.	Colon cancer, lymphoma	Suppressive	cGAS-STING activation	DCs, CD8^+^ T cells	(12)	Murine models
*Lachnoclostridium* spp.	Cutaneous melanoma	Suppressive	Elevated chemokines → increased CD8^+^ T cell infiltration	CD8^+^ T cells	(75)	Patient cohorts
*Helicobacter hepaticus*	Colon cancer	Suppressive	Activates T follicular helper cells → tertiary lymphoid structures	Tfh, B cells, NK cells	(76)	Murine models
*Cutibacterium acnes*	Various (syngeneic tumors)	Suppressive	Immune-contexture remodeling	–	(74)	Syngeneic tumor models

## 3 Harnessing live modified bacteria for cancer therapy

A conceptual distinction: Resident microbiota versus therapeutic bacterial vectors. Before discussing engineered bacterial platforms, it is essential to distinguish two biologically and therapeutically distinct entities. Naturally occurring intratumoral bacteria colonize tumors sporadically and exert context-dependent pro- or anti-tumor effects (discussed in Section 2). Exogenously administered, genetically engineered bacteria are deliberately introduced as therapeutic agents. While both can modulate the TME, they differ fundamentally in origin, genetic programmability, safety profiles, and translational pathways. Natural bacteria are not optimized for therapy—they may carry virulence factors, cannot be controlled for proliferation or payload expression, and vary unpredictably among patients. Engineered bacteria are attenuated, armed with defined genetic circuits, and administered at controlled doses. This section focuses exclusively on the latter. Where insights from natural tumor-resident microbes have inspired engineering strategies, such connections are explicitly noted.

To overcome constraints of standard anticancer regimens, investigators have engineered diverse bacterial species through virulence-attenuation modifications, redeploying them as immunotherapeutic vehicles or microbial delivery vectors. Preclinical investigations and human trials have validated *Streptococcus, Clostridium, Bifidobacterium, Listeria, Escherichia, Lactobacillus*, and *Salmonella* genera for these therapeutic platforms (22, 23, 26, 93).

### 3.1 Tumor targeting, penetration, and colonization by bacteria

**Figure 4:**
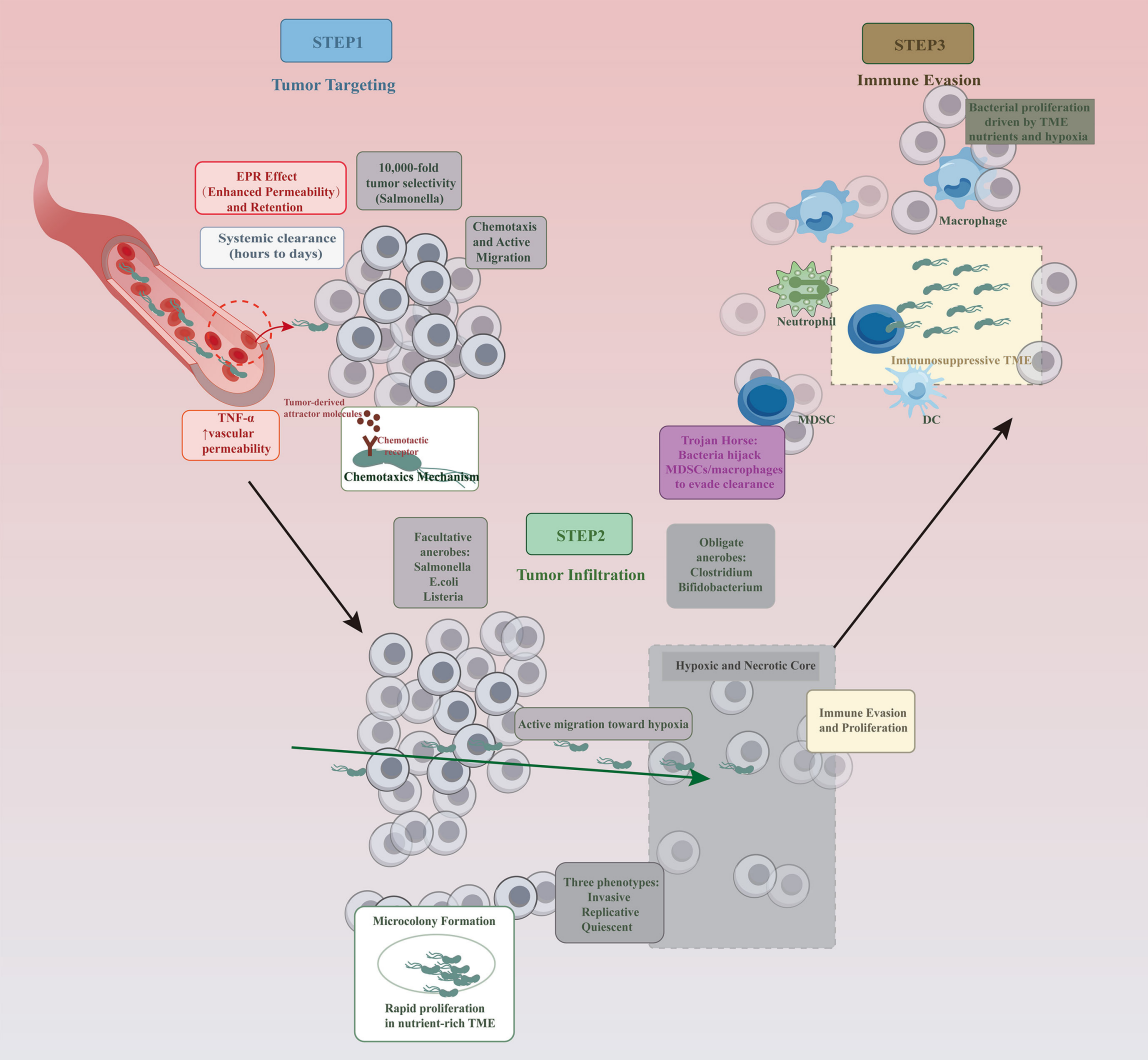
Mechanisms of tumor targeting, colonization, and immune evasion by therapeutic bacteria. Tumors possess a distinct microenvironment that differs significantly from most non-malignant tissues, making them preferential targets for certain bacteria - a trait that can be leveraged for bacteria-mediated cancer immunotherapy. In contrast, the TME often restricts the effectiveness of conventional cancer therapies, including other immunotherapies. The hypoxic regions and necrotic core of tumors create favorable conditions for both obligate and facultative anaerobes (95). Certain bacteria, such as *Clostridium* and *Bifidobacterium* species, are obligate anaerobes that thrive in oxygen-poor conditions and thus accumulate in Tumor hypoxic regions (96). Germinated *Clostridium* species have also been found near neoangiogenic vessels and in non-necrotic microinvasive lesions, indicating that these structures offer a suitable hypoxic and unique biochemical environment for bacterial colonization (97). As for facultative anaerobes like *Salmonella*, *Escherichia*, and *Listeria* species, their selective tumor targeting occurs through multiple mechanisms (26, 98). These include entrapment in the disordered tumor vasculature (99), chemotaxis toward tumor-released compounds (100), and preferential growth in tumor tissues due to favorable metabolic conditions (101). Pro-inflammatory cytokines like TNF can boost local blood flow, aiding bacterial infiltration into tumors (102). Bacterial chemotaxis is guided by receptors such as aspartate, ribose-galactose, and serine receptors, which direct *Salmonella* species to different tumor areas based on their nutritional status (103). *Salmonella* strain A1-R, a leucine and arginine auxotroph, thrives in the nutrient-rich TME but not in non-malignant tissues with low levels of these nutrients (101). *Listeria* species possess a unique tumor-targeting mechanism: they can infect antigen-presenting cells like monocytes, macrophages, and DCs (104). For example, by infecting tumor-infiltrating MDSCs, *Listeria* species can be transported to the TME and then spread to cancer cells. Although these pathogenic bacteria would be rapidly eliminated in non-malignant tissues, they can escape immune clearance by residing within MDSCs in the immunosuppressive TME (105). Created with Adobe Illustrator 2023.

Upon systemic delivery, high bacterial loads are transiently observed in the bloodstream and reticuloendothelial organs. Nevertheless, systemic clearance rapidly depletes bacteria from circulation and peripheral tissues (within hours to days), though their capacity to exploit tumor-selective homing mechanisms enables preferential colonization of solid tumors (94) (**Figure 4**). Tumor infiltration is mediated by bacterial transit through the hyperpermeable vasculature characteristic of the TME (25, 106). Critically, motile bacteria exhibit active migration toward hypoxic tumor cores rather than passive perivascular accumulation—a distinction from conventional drug diffusion dynamics (107). This directional tropism is driven by bacterial chemoreception of cancer cell death metabolites, which engage cognate receptors on bacterial surfaces (100, 103).

Upon intratumoral entry, bacterial proliferation establishes sustained colonization within neoplastic lesions (94). TME provides biochemical and structural conditions that selectively favor bacterial replication and persistence over non-malignant tissues (108). Notably, attenuated *Salmonella* serovars exhibit preferential tropism, achieving tumor-to-normal tissue accumulation differentials exceeding 10,000-fold in preclinical models (109). Spatial analysis of bacteriological distribution relative to Tumor vasculature reveals that macrocolonies localize predominantly perivascularly yet occupy regions of reduced microvessel density (108). Conversely, the majority of bacteria generate microcolonies dispersed heterogeneously throughout the Tumor parenchyma irrespective of vascular proximity (108). Further characterization identified three discrete functional phenotypes among Tumor-colonizing *Salmonella*: invasive, replicative, and quiescent subpopulations. These findings suggest tumor-infiltrating bacteria predominantly engage in either efficient tissue penetration or rapid proliferation, with individual organisms rarely exhibiting high competency in both capacities concurrently (108).

Bacterial colonization dynamically remodels the TME through immunostimulatory cascades; paradoxically, the immunosuppressive milieu simultaneously protects bacteria from host defenses, promoting localized proliferation while restricting their spatial dissemination (110). Neutrophils critically enforce bacterial containment within demarcated Tumor regions (102, 110), and their depletion compromises territorial restriction, enabling widespread bacterial dissemination (110). Macrophage ablation similarly enhances intratumoral bacterial burden (111). Bacterially recruited neutrophils exhibit preferential perinecrotic localization (102, 112). Where pathogen contact triggers extrusion of neutrophil extracellular traps (NETs)—chromatin-based matrices impregnated with antimicrobial effectors (113), and these structures immobilize and neutralize bacteria, confining them to discrete microniches (113), thereby constituting a key mechanism for spatial containment within Tumors.

### 3.2 Mechanisms of therapeutic bacteria

Bifunctional therapeutic capabilities characterize tumor-homing bacteria: they exert direct oncolytic effects on malignant cells while concurrently initiating coordinated immune activation of innate and adaptive defense networks. Synthetically engineered variants further function as programmable platforms for spatiotemporally controlled delivery of diverse therapeutic cargoes, substantially augmenting their translational utility in oncology (22).

#### 3.2.1 Direct cancer cell killing

Oncotic eradication by intratumoral bacteria operates through multimodal pathways. Proliferative colonization within neoplastic niches induces nutrient deprivation, provoking metabolic catastrophe and subsequent necrosis-driven cytotoxicity (95). This bioenergetic perturbation amplifies tumor cell vulnerability and enhances therapeutic outcomes (95). Intracellular *Salmonella* serovars trigger programmed cell death cascades (apoptosis/autophagy) via homeostasis disruption, processes mechanistically coupled to immunostimulatory cascades—including pro-inflammatory cytokine secretion and immune cell recruitment—that collectively establish an immunogenic TME (114, 115). Flagellin-mediated TLR5 engagement directly suppresses neoplastic proliferation while reprogramming anti-tumor immunity (116). *Listeria monocytogenes* induce subcellular organelle disruption in infected carcinoma cells through NADPH oxidase potentiation and calcium dysregulation, driving lethal oxidative burst (117). The auxotrophic strain *S. Typhimurium* A1-R, genetically constrained by leucine/arginine dependence, selectively propagates in tumor ecosystems and accelerates cell cycle transit (G0-G1→S-G2/M), sensitizing malignancies to cytotoxic agents (118, 119). Genetic circuitry enables bacterial synthesis of membrane-compromising cytotoxins (e.g., pore-forming cytolysin A), which disintegrate metabolic flux and induce apoptotic cascades in murine models (26, 120). Recombinant *Listeria* secreting listeriolysin O (LLO) similarly perforates plasma membranes, initiating osmotic lysis (121). Secretion systems delivering extrinsic apoptosis-inducing ligands (FASL/TRAIL/TNF) preferentially activate death receptors on malignant cells, orchestrating caspase-mediated elimination (122–124). Metabolically armed strains (e.g., L-asparaginase-expressing *Salmonella*) catalyze local depletion of asparagine, disabling biosynthetic pathways essential for tumor survival (125). Anti-angiogenic biofactories producing matricryptic peptides (Tum-5/thrombospondin-1) suppress neovascularization, curtailing xenograft progression in hepatic and dermal malignancies (109, 126, 127).

Genetically programmed bacteria engineered to express enzymatic prodrug conversion systems—including cytosine deaminase, HSV-1 thymidine kinase, and β-glucuronidase—significantly inhibit neoplastic progression in murine tumor models. These microbial platforms catalyze the biotransformation of inert prodrugs (5-fluorocytosine, ganciclovir, and 9-aminocamptothecin glucuronide) into their bioactive metabolites (5-fluorouracil, ganciclovir-3-phosphate, and 9-aminocamptothecin), thereby achieving spatiotemporal specificity in chemotherapeutic activation while mitigating systemic toxicity (128–130). The *Salmonella Typhimurium* variant VNP20009, synthetically modified to express carboxypeptidase G2, synergizes with prodrug substrates to elicit potent tumor regression, leveraging bacterial tropism for localized enzymatic activation within the TME (131).

Bacteria-Mediated Genetic Delivery: Bacterial vectors enable eukaryotic transgene expression via two distinct intracellular trafficking pathways: Cytosolic escape route: Invasive bacteria evade endosomal compartments, undergoing lytic release of plasmids that subsequently translocate to the nucleus; Vacuolar persistence route: Plasmid liberation occurs following bacterial metabolic exhaustion within endosomes, with nuclear uptake mediated by vesicular membrane traversal. Both pathways facilitate nuclear plasmid localization under eukaryotic transcriptional regulation (132). Recombinant *Salmonella choleraesuis* strains encoding anti-angiogenic transgenes (endostatin or thrombospondin-1) suppress neovascularization in preclinical models by paracrine factor secretion, disrupting tumor vascularization (109, 133). Similarly, telomerase-promoter-driven co-expression of TRAIL and SMAC (second mitochondria-derived activator of caspases) by *Salmonella* vectors induces caspase-dependent apoptosis exclusively in malignant cells, extending survival in tumor-bearing mice (134). Transcriptional Silencing via Bacterial Vectors: Salmonella-facilitated delivery of shRNA-encoding plasmids targeting oncogenic transcripts (*Stat3/Bcl-2*) enhances survival in murine models of prostate adenocarcinoma and melanoma by suppressing proliferative signaling and restoring apoptotic susceptibility. This approach leverages the ability of attenuated *Salmonella* strains to specifically deliver shRNA-expressing plasmids into tumor cells, thereby inducing RNA interference and selectively silencing oncogenic pathways. The targeted delivery of these plasmids by *Salmonella* ensures efficient gene silencing within the TME, highlighting the therapeutic potential of this strategy for cancer treatment (135, 136).

#### 3.2.2 Activation of immune cells

Bacteria inherently mediate immune activation and can be reprogrammed as modular bioplatforms for spatiotemporally controlled delivery of immunostimulatory agents, including cytokines, heterologous immune modulators (e.g., flagellin derivatives), TAAs, and RNA-based therapeutics (e.g., shRNAs/siRNAs) targeting immunosuppressive pathways within the TME (137–146). Upon colonization, bacteria elicit potent innate immunity, driving recruitment and functional polarization of neutrophils, DCs, macrophages, and T lymphocytes (147, 148). For instance, attenuated *Salmonella Typhimurium* strains activate innate immune cells to secrete pro-inflammatory mediators (IL-1β, TNF, IFNγ)—cytokines with direct oncolytic activity that additionally reprogram immunologically “cold” tumors into “hot” lesions. These cytokines not only exhibit direct anti-tumor activity against cancer cells but also promote inflammation, effectively converting immune “cold” tumors into “hot” tumors (149, 150). Lipopolysaccharide (LPS) engagement of TLR4 mediates inflammasome assembly, augmenting macrophage-dependent IL-1β biosynthesis and subsequent immunogenic phagocytosis of *Salmonella*-invaded malignant cells (150, 151). Concurrently, LPS drives TNF-dependent disruption of tumor vascular integrity (93). Bacterial colonization reprograms the functional landscape of NK cells within the TME, enhancing their cytolytic frequency and activity (152). Notably, flagellin-bearing CD11c^+^ cells evoke Toll-like receptor TLR-independent NK cell priming via IL-18/IL-18R-MYD88 signaling cascades, potentiating IFNγ production (153). Synthetic bacterial chassis engineered to secrete IL-18 or deliver endoglin DNA vaccines amplify tumor-infiltrating NK and T cell cytotoxicity in preclinical models, highlighting the dual-pathway immunomodulatory capacity of microbial vectors to concomitantly enhance innate and adaptive anti-tumor effector functions (154, 155). Further, STING pathway potentiation via *Escherichia coli* Nissle 1917 (EcN)-mediated delivery of cyclic di-AMP enhances DCs antigen presentation and type I interferon secretion (156). This signaling axis culminates in clonal T cell expansion and durable anti-tumor immunity in murine systems (156). The engineered EcN strain serves as a versatile platform for targeted immunotherapeutic interventions, leveraging its ability to modulate innate and adaptive immune pathways within the TME (156). Mechanistic insight into IL-10R-mediated immunomodulation: This investigation further elucidates how hysteresis-driven IL-10 receptor (IL-10R) dynamics within the TME establish a self-amplifying regulatory circuit that: (i) enhances alveolar macrophage (AM)-derived IL-10 biosynthesis, (ii) confers phagocytic evasion against tumor-associated neutrophils (TANs), and (iii) reinvigorates tumor-resident CD8^+^ T cell effector functions—collectively enabling selective expansion of synthetically modified *Salmonella* strains while orchestrating anti-tumor immunity (157).

BCIT predominantly depend on cytotoxic CD8^+^ T lymphocyte activation, pivotal effectors in anti-tumor immunity. *Salmonella* and *Listeria* species enhance intratumoral CD8^+^ T cell accumulation through chemokine upregulation driven by crosstalk with innate immune elements in the TME (150, 158, 159). Beyond direct oncolytic effects, *Clostridium novyi-NT* mediates indirect tumor suppression via TME-localized secretion of pro-inflammatory mediators including interleukin-6 (IL-6), chemokine (C-X-C motif), ligand 2 (CXCL2), and granulocyte colony-stimulating factor (G-CSF) (112). Genetically engineered *S. Typhimurium* strains, designed to secrete cytokines such as IL-2 (160), IL-15 (161), IL-18 (154), LIGHT, and CCL21 (162), have demonstrated the capacity to activate CD8^+^ T cells and elicit the production of pro-inflammatory cytokines, including IL-1β, IFNγ, and TNF. This immunomodulatory activity has been shown to enhance anti-tumor efficacy across multiple murine models. Furthermore, EcN and other engineered *E. coli* strains have been utilized to deliver nanobodies targeting immune checkpoints such as PD-L1 and CTLA-4 (163) or CD47 (164). These engineered systems have demonstrated the ability to induce sustained tumor regression and systemic anti-tumor immunity in preclinical murine models.

Moreover, bacteria have been designed to deliver TAAs for the purpose of eliciting tumor-specific CD8^+^ T cell activation. The TAAs targeted in these studies include prostate-specific antigen (140), CD24 (165), TRP2 (166), NY-ESO1 (141, 167), and VEGFR2 (168). *Listeria* preferentially target antigen-presenting cells, leading to increased MHC I presentation of epitopes from TAAs, including MAGE-B (117), prostatic acid phosphatase (169), and AH1 (an immunodominant antigen of the endogenous retroviral gp70 expressed in mouse CT26 colon cancer and 4T1 mammary cancer cells) (170). Multiple approaches have been developed to engineer *Salmonella* for tumor-associated antigen (TAA) delivery, including Direct intracellular release of TAAs (for example, the gastric cancer-associated antigen MG7-Ag) into infected cells (for example, via bacterial lysis) (171); Intracellular delivery of TAAs through fusion with type III secretion system (T3SS) proteins (for example, NY-ESO1, TRP2, or SopE) (141, 166); Surface display of TAAs on bacterial fimbriae proteins (for example, by inserting NY-ESO-1 epitopes into the fimbriae gene *AgfA*) *(*167).

The activation of the immune system through bacterial immunostimulation has a significant impact on the development of memory T (T_m_) cells, which is crucial for sustaining long-term anti-Tumor immunity and reducing the likelihood of tumor recurrence or metastasis (148, 170). T_m_ cells are categorized into two subtypes: effector memory (T_em_) and central memory (T_cm_) cells (148). T_em_ cells are predominantly found in peripheral non-lymphoid tissues such as the blood, while T_cm_ cells are usually located in lymphoid organs like the spleen (148). T_cm_ cells exhibit superior anti-tumor effectiveness compared to T_em_ cells, which is due to their higher production of inflammatory cytokines and their tendency to migrate to lymph nodes (172). A study comparing T_m_ cell induction using ovalbumin-delivering *S. Typhimurium* and *L. monocytogenes* revealed that *Listeria* primarily induces T_cm_ cells, while *Salmonella* mainly stimulates T_em_ cells (173). The induction of T_cm_ cells by *Listeria* was linked to enhanced anti-Tumor immunity in a B16-OVA melanoma mouse model (173). In another study, the delivery of the *L. monocytogenes* p60 protein via the T3SS of *Salmonella* led to the effective activation of CD8^+^ Tcm and CD8^+^ T_em_ cells specific for this antigen in the spleen, thereby providing mice with immunity against aggressive p60-transfected fibrosarcoma (174). In a breast cancer liver metastasis mouse model, bacterial vaccination was found to increase the proportion of CD4^+^ T helper cells and DCs in the liver and its draining lymph nodes, which was associated with enhanced IFNγ-mediated tumor-specific CD8^+^ T cell activity (175). DCs can engulf apoptotic bodies released from dying macrophages infected with bacteria and subsequently present bacterial antigens, which may include transgenic human TAAs, to T cells (176). The activation state of DCs is likely to be critical for the therapeutic efficacy of bacterial vaccines, as immature DCs are less efficient at cross-presenting antigens than mature DCs (177). It is also worth noting that *Salmonella* infection has been shown to induce the expression of connexin 43 in melanoma cells, promoting the formation of gap junctions between cancer cells and DCs and thereby enhancing the transfer and cross-presentation of preprocessed tumor antigens (178).

Specific bacterial strains actively combat immunosuppressive elements within the TME. Notably, the highly immunogenic *Salmonella* Typhi variant CVD 915 augments anti-tumor immunity by inhibiting T_reg_ cell function while concurrently enhancing the activity of IFNγ-producing CD4^+^ and CD8^+^ T lymphocytes in tumor-draining lymph nodes (147). Similarly, attenuated *Listeria* monocytogenes (deficient in ΔactA/ΔinlB) reduces T_reg_ cell prevalence and elevates the CD8^+^ T cell to T_reg_ cell ratio in the TME of CT26-bearing mice (159). Moreover, *L. monocytogenes* engineered to express LLO curtails the frequency and/or suppressive capacity of both MDSCs and T_reg_ cells. This strain additionally reprograms MDSCs to produce immunostimulatory interleukin-12 (IL-12), consequently amplifying the anti-tumor immune response across multiple preclinical models (105, 179).

Tumor-associated macrophages (TAMs) within the TME commonly polarize toward an immunosuppressive M2 state. Preclinical studies demonstrate that *Salmonella Typhimurium* modified to express heterologous flagellin B (FlaB) from Vibrio vulnificus, as well as attenuated *Listeria monocytogenes*, exert anti-tumor effects by inducing a phenotypic shift from immunosuppressive M2 to pro-inflammatory M1 macrophages in the TME (139, 180). Engineered *S. Typhimurium* secreting FlaB–IL-15 fusion constructs further catalyze M2-to-M1 macrophage conversion while concurrently enhancing proliferation and activation of adaptive and innate lymphoid populations within the TME (161). Similarly, attenuated *L. monocytogenes* expressing the AH1 epitope reprograms TAMs in CT26 tumor models through a mechanism dependent on interferon-gamma (IFNγ) secretion by tumor-specific CD8^+^ T cells, ultimately promoting tumor clearance (159).

Bacterial vectors have been employed to deliver short hairpin RNAs (shRNAs) and small interfering RNAs (siRNAs) targeting immunosuppressive pathways (142–145, 181). Notably, indoleamine 2,3-dioxygenase (IDO) enzymes—catalyzing the initial and rate-limiting conversion of tryptophan to kynurenine—are commonly overexpressed in malignancies (143). This enzymatic activity depletes local tryptophan pools within the TME, inhibiting T-cell activation and fostering immune tolerance (143). *Salmonella Typhimurium* engineered to deliver IDO-specific shRNAs suppresses IDO expression, enhances antigen presentation, diminishes T_reg_ cell frequency, and elevates DC, macrophage, and neutrophil infiltration. These collective modifications enhance anti-tumor immunity and improve immune checkpoint inhibitor (ICI) efficacy across preclinical cancer models (142–144). Furthermore, *Salmonella*-mediated PD-1-targeted siRNA delivery demonstrates significant anti-tumor activity in murine colon cancer and melanoma, particularly when combined with nifuroxazide (STAT3 inhibitor) or pimozide (dopamine antagonist) (145, 181).

Bacterial immunomodulation extends to metabolic reprogramming. TME frequently exhibits depletion of key immunomodulatory metabolites (e.g., L-arginine, tryptophan) and accumulation of immunosuppressive metabolites (e.g., lactate, kynurenine). Engineered bacteria can act as living metabolic regulators that precisely intercept or restore these metabolic pathways. For example, L-arginine depletion, caused by upregulated arginase in malignant cells and immunosuppressive populations (MDSCs, macrophages, T_reg_ cells)program (182, 183), compromises T-cell functionality (182, 183). To counter this, a metabolically engineered EcN strain was designed to overproduce L-arginine via feedback-resistant biosynthesis from ammonia—a metabolic byproduct (184). Tumor colonization by this engineered bacterium amplifies tumor-infiltrating T lymphocytes and potently synergizes with PD-L1-targeted ICIs (184). Similarly, tryptophan catabolism by IDO generates kynurenine, which promotes immunosuppression. Engineered *Salmonella* delivering IDO-specific shRNA not only reduces kynurenine but also reshapes the immune contexture. Beyond amino acid metabolism, bacterial modulation of lactate and short-chain fatty acids represents an emerging frontier. For instance, lactate-consuming engineered *E. coli* have been shown to alleviate TME acidosis and enhance T cell function in preclinical models. Future efforts should focus on high-throughput screening of bacterial metabolites that restore anti-tumor immunity, coupled with synthetic biology to construct regulatable metabolic circuits. Such “living metabolic regulators” offer a paradigm shift in cancer immunotherapy by reprogramming the TME at the metabolic level.

#### 3.2.3 Clinical landscape of bacteria-mediated cancer immunotherapy

Several bacterial platforms have advanced to clinical trials. The attenuated Salmonella Typhimurium strain VNP20009, engineered to express cytosine deaminase, was evaluated in Phase I trials for refractory cancer patients but showed modest antitumor activity and dose-limiting toxicity (185). Clostridium novyi-NT spores have been tested in patients with advanced solid tumors via intratumoral injection; objective responses and tumor regression were observed in a subset of patients, accompanied by manageable local inflammatory reactions (186). *Listeria* monocytogenes-based vaccines, such as ADXS11-001 (targeting HPV-associated cancers), have completed Phase II trials, demonstrating safety and immunological activity, though efficacy varied (187). More recently, an engineered *E. coli Nissle* 1917 strain delivering STING agonist (SYNB1891) entered Phase I trial for advanced solid tumors and lymphomas (NCT04167137) (188). These clinical experiences highlight both the potential and the challenges of BCIT, including optimal dosing, off-target toxicity, and the need for patient stratification (189). While no BCIT has yet received regulatory approval, ongoing and future trials will clarify their therapeutic niche, likely in combination with immune checkpoint inhibitors.

## 4 Strategies for reprogramming bacteria

### 4.1 Controlling gene regulation

**Figure 5:**
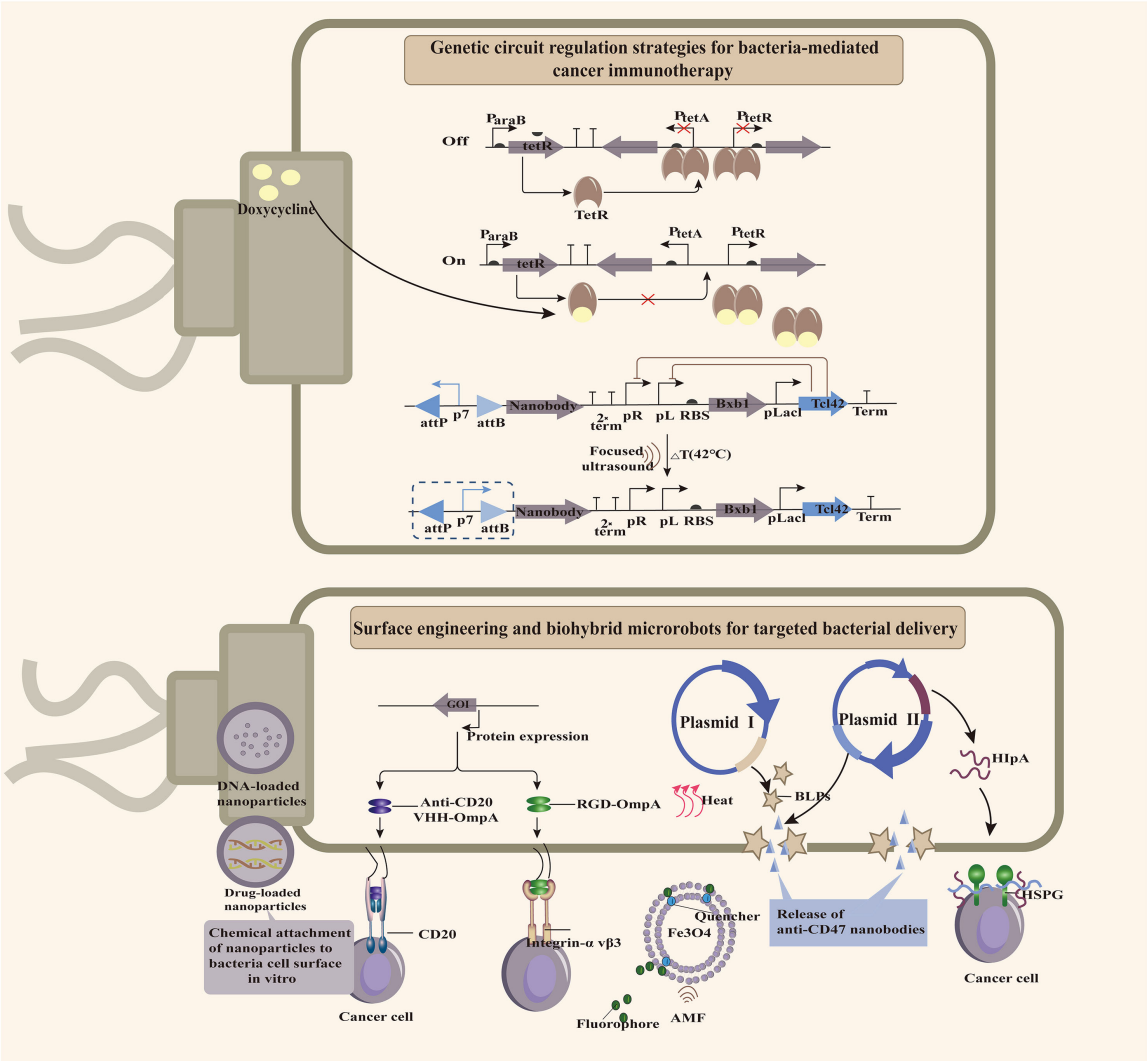
Engineering strategies for bacteria-mediated cancer immunotherapy. This figure presents four complementary approaches for reprogramming therapeutic bacteria, organized into two thematic sections (Top and Bottom). Top – Genetic circuit regulation: The left panel illustrates a doxycycline-inducible system, where TetR repressor blocks payload transcription until doxycycline administration relieves repression, enabling titratable transgene expression. The right panel depicts a thermal memory switch, where focused ultrasound-induced heating (42 °C) inactivates the thermolabile repressor Tcl42, activates Bxb1 integrase, and drives irreversible inversion of attP/attB sites, leading to sustained nanobody expression. Bottom – Surface engineering and biohybrid microrobots: The left panel shows surface display of targeting ligands (RGD peptides, anti-CD20 VHH antibodies) on outer membrane protein A (OmpA) for enhanced tumor specificity, as well as nanoparticle coating for drug/DNA loading. The right panel illustrates biohybrid microrobots, where bacteria conjugated with magnetic nanoparticles respond to alternating magnetic fields (AMF), generating localized heat that triggers bacterial lysis and on-demand release of anti-CD47 nanobodies. Created with Adobe Illustrator 2023.

Transforming bacteria into effective BCIT agents necessitates advanced genetic reprogramming (190, 191) (**Figure 5**). Precision-tuning therapeutic transgene expression maximizes clinical benefits while minimizing off-target toxicity. Engineered transcriptional control systems—utilizing pathogen-inducible promoters to govern therapeutic cargo genes—enable spatiotemporal precision in payload delivery *in vivo*. These regulatory architectures fall into three principal categories: exogenous inducer-responsive systems, endogenous stimulus-activated circuits, and synthetically designed genetic networks (23, 24, 192, 193).

#### 4.1.1 External triggering

Multiple chassis-compatible promoter systems responsive to administered small molecules have been developed for titratable transgene expression. Key examples encompass: The AraC-regulated araBAD promoter induced by L-arabinose (139, 161, 194); lacI-repressible promoters (Ptac, Ptrc) activated by lactose analogs (195, 196); The XylS2-dependent Pm promoter triggered by acetylsalicylic acid (197). Additionally, bidirectional tetracycline-responsive promoters permit stoichiometric co-expression of multiple payloads when induced by clinically approved tetracycline-class antibiotics (e.g., doxycycline) (198, 199) (**Figure 5**). However, clinical translation of chemically inducible systems faces challenges including inducer toxicity and promoter leakage (200).

Externally applied physical stimuli offer alternative induction modalities. Photonic stimulation using blue light-responsive promoters (e.g., pDawn) enables spatial control of therapeutic protein production, as demonstrated by tumor necrosis factor-related apoptosis-inducing ligand (TRAIL) expression for selective cytotoxicity (201). Thermosensitive (e.g., pR-pL tandem) and radiation-inducible (e.g., RecA) (122) promoters further expand the BCIT toolkit. Nevertheless, physical induction strategies contend with limitations including restricted tissue penetrance, off-target activation, and suboptimal spatial resolution (202).

#### 4.1.2 Internal triggering and quorum sensing

Endogenous stimuli characteristic of the TME can activate engineered bacterial expression platforms. Promoters responsive to pathological conditions—including the oxygen-depletion inducible FF + 20* (203), acid-triggered adiA (204), and nitric oxide (NO)-detecting genetic circuits (205)—have been successfully leveraged to initiate therapeutic transgene expression under tumor-specific conditions.

In addition, engineered bacteria can be controlled by quorum sensing, a phenomenon where signaling molecules monitor population density changes (25, 206). Since certain bacteria selectively colonize tumors, their quorum sensing systems can trigger therapeutic payload release specifically in tumors, reducing harm to non-malignant tissues (191, 207). Initial preclinical validation demonstrated *Salmonella*’s density-responsive quorum sensing circuitry could restrict cargo protein (e.g., GFP) expression exclusively to high-density bacterial clusters within tumors (208). Subsequent engineering of EcN incorporated a phage-derived lysis gene governed by an acyl-homoserine lactone-responsive (*luxI/luxR*) quorum sensing module. This design enables programmed bacterial lysis and localized release of immunotherapeutic payloads—including anti-PD-L1 and anti-CTLA-4 nanobodies—upon reaching threshold cellular density within the TME (163).

#### 4.1.3 Synthetic gene circuits

Synthetic biology and genetic engineering fundamentally rely on DNA manipulation. Historically, therapeutic bacterial constructs primarily utilized constitutive or minimally regulated promoters for transgene expression. Contemporary advances in synthetic genetic circuitry now permit refined transcriptional governance, enabling novel functionalities and advanced control modalities (193, 209–211).

Genetically encoded memory circuits generate persistent cellular outputs following transient stimuli, effectively converting ephemeral signals into durable biological responses. For instance, the thermolabile repressor Tcl42 mediates a memory circuit directing EcN to express immune checkpoint nanobodies (anti-PD-L1/anti-CTLA-4) (212). Under physiological conditions, Tcl42 binds pL promoters to suppress Bxb1 integrase transcription (212). However, focused ultrasound-mediated thermal denaturation of Tcl42 induces Bxb1 expression, catalyzing irreversible inversion of attP/attB sequences. This genomic rearrangement reorients the embedded P7 promoter to drive sustained nanobody production, establishing a heat-actuated genetic switch (212). Locally secreted nanobodies enhance T-cell function within the TME and inhibit tumor progression in murine models (212) (**Figure 5**). An alternative memory architecture employs a dual-plasmid system: Plasmid 1 encodes fimE recombinase under doxycycline- or NO-inducible control (198, 205); Plasmid 2 houses therapeutic genes downstream of a constitutive promoter flanked by inverted repeats (198, 205). Inducer-mediated fimE activation permanently inverts the constitutive promoter, transitioning the system from silent to active states to enable persistent transgene expression (198, 205).

Boolean logic gates further permit signal-integrated output generation, producing defined responses only upon receiving specific combinatorial inputs (213). Similarly, tumor-microenvironment-responsive circuits couple bacterial proliferation to pathological biomarkers (e.g., hypoxia, lactate accumulation, acidic pH) via cognate promoters, confining transgene activity to neoplastic sites (214). However, to enhance specificity given the non-exclusive nature of these biomarkers, dual-input “AND” gates integrating hypoxia-lactate biosensors have been engineered to improve tumoral targeting precision (215). These multiplexed genetic circuits may improve the precision of bacterial targeting and localization within tumors (213, 215).

Sophisticated synthetic genetic circuits can dynamically modify bacterial membrane composition in response to extrinsic cues. For instance, using a lactose-inducible promoter can temporarily activate the expression of capsular polysaccharide, a surface defense layer in certain bacteria (216). Preclinical studies demonstrate this approach significantly elevated the maximum tolerated intravenous dose of EcN by an order of magnitude, resulting in enhanced tumoral localization and therapeutic outcomes (216).

### 4.2 Bacterial surface engineering

#### 4.2.1 Surface display

Surface engineering approaches further enable bacterial membrane modifications (**Figure 5**). For example, ice nucleation proteins (INPs) facilitate the anchoring of exogenous proteins onto microbial surfaces (217, 218). Fusion of HlpA with an INP motif permits its display on EcN, augmenting adhesion to heparan sulfate proteoglycans abundant in CRC cells. This engineered EcN strain additionally expresses myrosinase, catalyzing the transformation of plant-derived glucosinolates into the oncolytic metabolite sulforaphane (219). Parallel strategies include engineering *Salmonella Typhimurium* to present integrin-binding RGD motifs within the extracellular domain of OmpA, heightening specificity for αvβ3 integrin-overexpressing malignancies (220) (**Figure 5**). Additionally, genetic fusions of OmpA with anti-carcinoembryonic antigen scFv fragments (221) or anti-CD20 camelid single-domain (VHH) antibodies (222) similarly direct *Salmonella* tropism toward antigen-expressing neoplasms (**Figure 5**).

Aptamers—synthetic oligonucleotide ligands engineered for selective molecular recognition—demonstrate superior target-binding specificity and affinity compared to conventional antibodies or ligands (223). Their compact architecture and modular design enhance versatility for therapeutic applications (223). Bacterial surface conjugation via cytocompatible amidation significantly improves tumor-specific localization post-systemic delivery (224). Illustratively, AS1411 aptamer-conjugated attenuated *Salmonella* targets nucleolin-overexpressing carcinomas (e.g., 4T1 mammary tumors), eliciting potent intratumoral immune activation and anti-tumor efficacy in murine models (224).

#### 4.2.2 Bacterial coating with nanoparticles

Therapeutic bacteria can also be engineered with nanoparticles coating their membrane to enhance tumor colonization (225) (**Figure 5**). Nanoparticle-functionalized bacteria represent promising drug delivery platforms. Concurrent engineering of bacterial and nanomaterial components affords versatile payload incorporation capabilities and improved tumor penetration, overcoming constraints associated with conventional nanotherapeutic systems (226). Preclinical investigations demonstrate the biomineralization of gold nanoparticles on tumor-homing *E. coli* MG1655 engineered to secrete TNF via a thermosensitive Tcl42-regulated circuit. Following oral administration, exogenous near-infrared irradiation of tumors induces photothermal activation of nanoparticles, stimulating localized TNF production within the TME (227). Furthermore, therapeutic agents including paclitaxel and perfluorohexane have been co-loaded into nanoparticles electrostatically conjugated to bacteria, facilitating targeted delivery to hypoxic neoplastic regions (228). *Salmonella* coated with cationic polymer-plasmid nanoparticle complexes enables oral administration of VEGFR2 DNA vaccines (229). This strategy reprograms the immunosuppressive TME to promote T-cell activation and inhibit neoplastic progression in murine models. Additionally, synergistic application of radiotherapy with *Salmonella* carrying antigen-trapping polymeric nanocarriers enhances the peritumoral accumulation of TAAs, improving DC-mediated antigen presentation and generating abscopal anti-tumor responses (230).

### 4.3 Biological robots for drug delivery

*Escherichia coli*, Serratia marcescens, and *Salmonella* Typhimurium serve as widely explored biological engines in the construction of biohybrid microswimmers designed for large-payload delivery. These autonomously motile systems, equipped with environmental sensing capabilities, represent ideal platforms for the active transport of therapeutic compounds (231, 232). Illustrating this approach, microswimmers engineered by conjugating doxorubicin-loaded microparticles incorporating magnetic nanoparticles to *E. coli* demonstrated magnetically directed anticancer agent delivery to mammary carcinoma cells under *in vitro* conditions (233). Furthermore, *E. coli* MG1655 has been harnessed to propel biohybrid microswimmers transporting erythrocytes co-loaded with doxorubicin and superparamagnetic iron oxide nanoparticles, where bacterial motility provides thrust and the nanoparticles facilitate magnetic steering (234). Similarly, microrobotic biohybrids integrating motile *E. coli* carriers bearing magnetic nanoparticles functionalized with both photothermal and chemotherapeutic agents exhibit navigational capacity through biological barriers, colonizing tumor spheroids via magnetic guidance before near-infrared-triggered payload release (235). A synergistic control strategy merging magnetic torque-based navigation with autonomous chemotactic motility has also been applied to enhance tumor infiltration by the intrinsically magnetotactic bacterium Magnetospirillum magneticum, resulting in improved neoplastic colonization in three-dimensional spheroid models (236). This versatile platform employs a uniform rotating magnetic field—clinically scalable for directional control without reliance on visual feedback.

AMF offers exceptional utility for manipulating tumor-colonizing bacteria, owing to their profound tissue penetration depth and favorable biocompatibility profile (237). AMF-responsive, tumor-homing bacterial constructs have been developed through conjugation of a Fe_3_O_4_@lipid nanocomposite to genetically modified *E. coli* BL21 constitutively expressing (HlpA; targeting heparan sulfate proteoglycans overexpressed in colorectal cancers) and anti-CD47 nanobodies (238). Beyond enabling magnetically guided tumor targeting, the integrated Fe_3_O_4_ nanoparticles function as a signal-decoding module, transducing magnetic energy into thermal energy. This localized heating activates a thermosensitive promoter-controlled genetic circuit encoding BLPs (signal processing module) and concurrently disrupts the temperature-responsive lipid monolayer, liberating fluorophores from quenchers to provide optical feedback on biorobot activation (signal feedback module). Consequently, AMF application permits spatiotemporally precise induction of bacterial lysis and therapeutic nanobody liberation, facilitating localized CD47 blockade within the TME (**Figure 5**). **Table 2** provides an overview of engineered bacterial platforms, including genetic modifications, payloads, control systems, and therapeutic outcomes.

**Table 2:** Engineered bacterial therapeutics for cancer immunotherapy: key features and representative examples.

Bacterial chassis	Genetic modification	Therapeutic payload	Induction/control system	Delivery route	Tumor model	Major therapeutic effect	References
*S. Typhimurium* VNP20009	Cytosine deaminase	5-FU (from 5-FC)	Constitutive	i.v.	Refractory cancer patients (Phase I)	Modest antitumor activity	(128)
*S. Typhimurium*	shRNA against IDO	IDO silencing	Constitutive	i.v.	CRC, melanoma	Suppressed tumor growth, enhanced ICI efficacy	(142–144)
*S. Typhimurium*	Flagellin B (FlaB)	Immunostimulant	L-arabinose-inducible	i.v.	Colon cancer, melanoma	M2→M1 macrophage conversion, T cell activation	(139)
*S. Typhimurium*	FlaB-IL-15 fusion	IL-15	L-arabinose-inducible	i.v.	Colon cancer	Enhanced NK and CD8^+^ T cell proliferation	(161)
*L. monocytogenes* ΔactA/ΔinlB	LLO + TAA (e.g., AH1, MAGE-B, PAP)	Antigen delivery	Constitutive	i.v.	Breast cancer, CRC, hepatic metastases	CD8^+^ T cell response, Treg reduction	(117, 159, 169, 170)
*E. coli* Nissle 1917 (EcN)	luxI/luxR quorum sensing + lysis gene	Anti-PD-L1, anti-CTLA-4 nanobodies	Population-density controlled	i.v.	CRC, melanoma	Local immune checkpoint blockade	(163)
*E. coli* Nissle 1917	Cyclic di-AMP	STING agonist	Constitutive	i.t.	Melanoma	DC activation, type I IFN, CD8^+^ T cell response	(156)
*E. coli* Nissle 1917	Feedback-resistant arginine biosynthesis	L-arginine	Constitutive	i.v.	Colon cancer, melanoma	Increased T cell infiltration, synergizes with anti-PD-L1	(184)
*E. coli *MG1655	Thermosensitive Tcl42 + TNF	TNF	Focused ultrasound (42 °C) + photothermal	Oral + NIR	Breast cancer	Localized TNF production	(227)
*E. coli* BL21	Fe_3_O_4_@lipid + thermosensitive promoter + HlpA	Anti-CD47 nanobody + BLPs	Alternating magnetic field (AMF)	i.v.	Breast cancer	AMF-triggered lysis, CD47 blockade	(238)
*S. Typhimurium*	RGD peptide on OmpA	– (targeting)	Constitutive	i.v.	Integrin αvβ3^+^ tumors	Enhanced tumor targeting	(220)
*S. Typhimurium*	Anti-CD20 VHH on OmpA	– (targeting)	Constitutive	i.v.	CD20^+^ lymphoma	Enhanced tumor targeting	(222)
Attenuated *Salmonella*	PD-1 siRNA	PD-1 silencing	Constitutive	i.v.	Colon cancer, melanoma	Enhanced anti-tumor effect with nifuroxazide/pimozide	(145, 181).

## 5 Considerations for future implementation of BCIT: challenges and opportunities

### 5.1 Major challenges

Several crucial considerations are necessary for the clinical translation and implementation of BCIT, despite significant advancements in their development. These include discrepancies between preclinical and initial clinical study results, along with other key challenges mentioned here.

First, the unclear mechanisms of BCIT hinder their development; exploring their main mechanisms is vital for improving therapeutic effects (239, 240). Moreover, the possible synergistic effects of combining BCIT with existing treatments need deeper research.

Second, BCIT may cause adverse effects seen in current immunotherapies, especially ICIs, such as systemic immune reactions, hypertension, and fatigue (241). Employing attenuated pathogens in BCIT introduces inherent toxicity hazards stemming from their residual pathogenic potential. Consequently, rigorous patient stratification is imperative. Systemically administered live bacteria pose elevated infection risks for immunocompromised individuals or those receiving immunosuppressive cytotoxic regimens. Predisposing factors—including cerebral abscesses, recent radiotherapy, or prosthetic joints/valves—may further facilitate ectopic bacterial proliferation (242–244).

Third, the pharmacokinetic profile of live bacteria diverges fundamentally from conventional pharmaceuticals, yielding non-linear dose-response dynamics. Therapeutic efficacy depends critically on the TME rather than solely on inoculum size. Furthermore, active bacterial replication within target lesions decouples the administered dose from the resultant therapeutic payload (25). Establishing correlations between optimized bacterial dosing, intratumoral pharmacokinetics, and specific cancer contexts—particularly for systemic BCIT—is therefore essential (23).

Fourth, conventional sterilization methods (e.g., heat or filtration) are incompatible with live biotherapeutics, rendering standard sterility assays inadequate. This presents substantial manufacturing challenges for producing clinical-grade products under Good Manufacturing Practice (GMP) standards (25). Additionally, existing regulatory frameworks for traditional drugs are ill-suited for evaluating self-replicating agents, necessitating collaborative engagement with regulatory agencies to develop appropriate guidelines.

Fifth, pivotal translational challenges—including intellectual property protection, scalable production, and product stability—demand resolution for successful BCIT implementation. Addressing these requires harmonized protocols and concerted efforts between developers, manufacturers, and regulators to facilitate clinical translation.

### 5.2 Bridging the gap: from intratumoral microbiome insights to next-generation BCIT

The preceding sections have described two parallel tracks: the dual roles of naturally occurring intratumoral bacteria in tumor immunity (Section 2) and the engineering of bacterial platforms for cancer therapy (Sections 3–4). A critical, yet underexplored, question is how knowledge from the first track can inform the rational design of the second. Here, we outline several promising strategies that directly translate microbiome insights into improved bacterial therapeutics.

#### 5.2.1 Exploiting molecular mimicry for enhanced t cell therapies

Tumor-specific antigens (TSAs) and tumor-associated antigens (TAAs) are classical targets for cancer immunotherapy. The discovery that peptides derived from commensal bacteria—such as those from *Bifidobacterium breve* (78) and prophage-containing *Enterococcus hirae*, share structural and linear homology with TSAs opens a new avenue. These microbial mimics can prime cross-reactive CD8^+^ T cells that recognize malignant cells. This insight can be harnessed in at least two ways. First, bacterial strains carrying such cross-reactive epitopes could be used as live vaccines to expand tumor-reactive T cell pools *in vivo*. Second, T cell receptors (TCRs) isolated from patients with favorable responses to BCIT or with high abundance of such bacterial strains could be sequenced and used to generate TCR-engineered T cells for adoptive transfer. Proof-of-concept studies in mouse models have validated this approach, but clinical translation requires systematic screening of patient-derived bacterial isolates for TSA-mimicking epitopes, followed by rigorous safety testing.

#### 5.2.2 Microbiome-informed payload selection

The composition of intratumoral microbiota varies substantially across patients and correlates with immunotherapy outcomes. For example, enrichment of Clostridium in melanoma tumors associates with ICI response, whereas Gardnerella vaginalis associates with non-response (13). In PDAC, Pseudoxanthomonas-Streptomyces-Saccharopolyspora-Bacillus consortia correlate with long-term survival (21). These signatures can guide the selection of bacterial chassis and therapeutic payloads for engineered BCIT. A patient whose tumor harbors Fusobacterium nucleatum, which upregulates PD-L1 (68, 69) and recruits MDSCs, might benefit from an engineered E. coli Nissle strain delivering anti-PD-L1 nanobodies combined with a payload that depletes MDSCs (e.g., IDO shRNA (142–144). Conversely, a patient with Bifidobacterium-enriched tumors, which activate STING signaling, might be a candidate for STING-agonist-delivering BCIT. This microbiome-informed personalization remains largely conceptual; prospective clinical trials are needed to validate whether matching BCIT design to the pre-treatment intratumoral microbiome improves outcomes.

#### 5.2.3 Artificial intelligence for integrating microbial and tumor data

The complexity of microbiome-host interactions exceeds intuitive human reasoning. Machine learning models have been successfully applied to predict cancer type from microbial signatures and to identify bacterial taxa associated with ICI response (245). For BCIT, AI could integrate: (i) the patient’s intratumoral and gut microbiome composition (from shotgun metagenomics), (ii) tumor genomic and transcriptomic profiles (e.g., neoantigen landscape, MHC expression, immune infiltration), and (iii) clinical variables (e.g., prior treatments, antibiotic exposure). The output would recommend an optimal bacterial chassis, genetic circuit, payload, and delivery route. A concrete workflow could be: patient data → AI classifier → strain/payload selection → predicted response → clinical validation. Feasibility studies are ongoing for cancer immunotherapy (246, 247), but dedicated BCIT-specific AI models have not yet been developed. High-quality, multi-omics datasets from BCIT trials will be a prerequisite.

#### 5.2.4 Collaborative trial design and regulatory considerations

Translating these personalized concepts into clinical practice requires infrastructure that spans multiple disciplines. We propose that future BCIT trials should: (i) pre-screen patients for intratumoral microbiota composition (e.g., via biopsy sequencing) as an enrollment criterion or stratification variable; (ii) bank bacterial isolates from trial participants to enable retrospective analysis of responder/non-responder microbial signatures; (iii) incorporate adaptive designs that allow assignment of BCIT variants (e.g., different payloads) based on real-time microbiome data. These recommendations are not yet standard but are within reach given current sequencing and bioinformatics capabilities. Regulatory agencies (e.g., FDA, EMA) have begun issuing guidance on live biotherapeutic products (239, 240); we encourage investigators to engage early with regulators to define product specifications for personalized BCIT.

#### 5.2.5 Outstanding questions and limitations

The strategies outlined above face substantial hurdles. First, the functional contribution of low-abundance intratumoral bacteria is difficult to assess; many detected organisms may be passengers rather than drivers. Second, causality cannot be inferred from correlation—microbiome signatures associated with response may be consequences, not causes, of effective immunity. Third, safety concerns remain: introducing engineered bacteria into patients with microbiome-altering conditions (e.g., inflammatory bowel disease) could provoke unpredictable outcomes. Fourth, the cost and turnaround time for personalized BCIT (including sequencing, AI analysis, and bacterial engineering) currently exceed those of standard therapies. Addressing these limitations will require iterative preclinical refinement and carefully designed early-phase trials.

In summary, the convergence of microbiome research, synthetic biology, and AI offers a rational pathway to next-generation BCIT. Rather than making speculative predictions about trial timelines, we emphasize that the immediate priorities should be (1): establishing standardized protocols for intratumoral microbiome sampling and analysis (2); building public databases linking microbial features to BCIT outcomes; and (3) developing modular, rapidly engineerable bacterial chassis. These foundational efforts will determine the pace of clinical translation.

## 6 Conclusions

**Figure 6:**
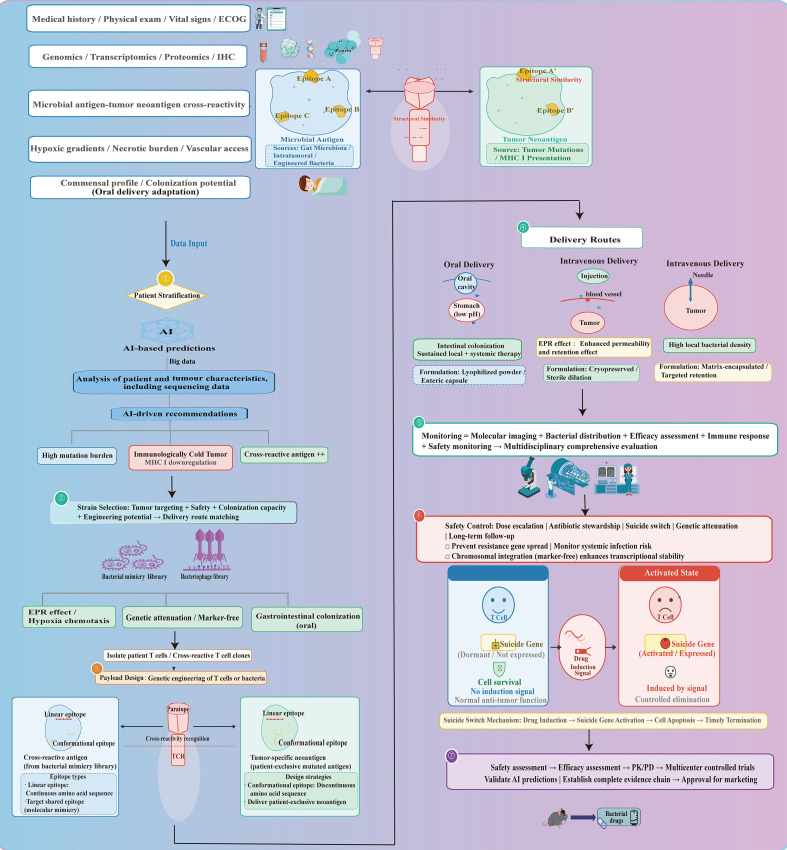
An AI-guided six-step pipeline for personalized bacteria-mediated cancer immunotherapy. This schematic outlines a comprehensive framework integrating multi-omics data, artificial intelligence, synthetic biology, and clinical trial design to translate BCIT into personalized therapeutic strategies. Step 1 – Patient Stratification: Patient eligibility is assessed using clinical data (history, physical exam, ECOG status), tumor multi-omics (genomics, transcriptomics, proteomics, IHC), hypoxia/necrotic burden imaging, vascular access evaluation, and gut microbiota profiling. Step 2 – AI-Driven Recommendation: Machine learning algorithms integrate big data to predict optimal therapeutic strategies based on tumor characteristics (mutation burden, MHC-I expression, immune infiltration) and commensal colonization potential, recommending the most suitable bacterial chassis, delivery route, and therapeutic payload. Step 3 – Epitope/Neoantigen Design: Two antigenic strategies are pursued: (i) patient-exclusive tumor-specific neoantigens (mutated antigens); and (ii) cross-reactive microbial epitopes (linear or conformational) selected from bacterial mimic libraries to enable T cell cross-reactivity via molecular mimicry. Step 4 – Engineering and Safety Control: Selected bacterial strains are genetically attenuated (marker-free chromosomal integration), engineered with therapeutic payloads, and equipped with inducible safety switches (e.g., suicide genes for timely termination) to prevent resistance gene spread and systemic infection risk. Step 5 – Real-Time Monitoring: Molecular imaging tracks bacterial biodistribution, therapeutic efficacy, immune responses, and safety parameters. A multidisciplinary team (oncologists, radiologists, infectious disease specialists) conducts comprehensive evaluations throughout the treatment course. Step 6 – Clinical Validation: AI-driven predictions are prospectively validated through multicenter controlled trials, with rigorous safety and efficacy assessments, PK/PD profiling, and long-term follow-up, ultimately establishing a complete evidence chain for regulatory approval. Abbreviations: ECOG, Eastern Cooperative Oncology Group; IHC, immunohistochemistry; MHC-I, major histocompatibility complex class I; PK/PD, pharmacokinetics/pharmacodynamics. Created with Adobe Illustrator 2023.

BCIT is an emerging oncology approach using bacteria’s unique properties to treat cancer. Historically noted for cancer regression post-infection, it gained scientific momentum in the early twentieth century. Recent molecular biology and technical advances in sequencing have revived interest, especially in intratumoral bacteria’s role within the TME. BCIT can overcome the immunosuppressive TME, aiding current immunotherapies. Combining BCIT with treatments like ICIs may produce synergistic effects. Ongoing clinical trials are assessing engineered bacterial strains for safety, efficacy, and administration methods, crucial steps for BCIT’ integration into standard cancer care. However, challenges like regulatory hurdles, limited patient acceptance, and safety concerns with genetically modified microorganisms persist. BCIT success hinges on overcoming these challenges, yet its potential benefits make it a valuable research and development area. Lastly, BCIT involves complex mechanisms, with current research predominantly concentrating on immune checkpoint molecules such as PD-1, CTLA-4, TIM-3, and LAG-3. Key considerations for the future clinical translation of BCIT, including molecular mimicry, AI-driven personalization, and multidisciplinary collaboration, are summarized in **Figure 6**.

## Glossary

AICD: Activation-induced cell death
ADCC: antibody-dependent cell-mediated cytotoxicity
TME: tumor microenvironment
NSCLC: non-small-cell lung cancer
ICIs: immune checkpoint inhibitors
BCIT: bacteria-mediated cancer immunotherapy
DDR: DNA damage response
ICB: immune checkpoint blockade
DSBs: DNA double-strand breaks
CRC: colorectal cancer
Bft: Bacteroides fragilis enterotoxin
SNHG17: small nuclear RNA host gene 17
CagA: cytotoxin-associated gene A
S. Typhimurium: Salmonella enterica serovar Typhimurium
S. Typhi: S. enterica serovar Typhi
NLR: nucleotide-binding oligomerization domain-like receptor
NK: natural killer
PDAC: pancreatic ductal adenocarcinoma
MDSCs: myeloid-derived suppressor cells
AhR: aryl hydrocarbon receptor
FMT: faecal microbiota transplantation, STING, sensing via the cGAS-interferon genes
DCs: dendritic cells
TAAs: tumor-associated antigens
TSAs: tumor-specific antigens
NETs: neutrophil extracellular traps
LLO: listeriolysin O
LPS: lipopolysaccharide
EcN: Escherichia coli Nissle 1917
IL-10R: IL-10 receptor
AM: alveolar macrophage
TANs: tumor-associated neutrophils
IL-6: interleukin-6
G-CSF: granulocyte colony-stimulating factor
T3SS: type III secretion system
IL-1: 2interleukin-12
TAMs: tumor-associated macrophages
FlaB: flagellin B
shRNAs: short hairpin RNAs
siRNAs: small interfering RNAs
IDO: indoleamine 2,3-dioxygenase
RGD: Arg-Gly-Asp
OmpA: outer membrane protein A
HlpA: histone-like protein A
HSPG: heparan sulfate proteoglycan
AMF: alternating magnetic field
BLPs: bacterial lysis proteins
TRAIL: tumor necrosis factor-related apoptosis-inducing ligand
NO: nitric oxide
INPs: ice nucleation proteins
VHH: anti-CD20 camelid single-domain
GMP: Good Manufacturing Practice
AI: artificial intelligence
